# Meeting of the Cook County Medical Society

**Published:** 1857-10

**Authors:** Thomas Bevan

**Affiliations:** Secretary, Chicago


### PROCEEDINGS OF MEDICAL SOCIETIES.

CHICAGO, JULY 7TH, 1857.

Cook County Medical Society met pursuant to notice, at the office of Dr. Chas. G. Smith.

Present—Drs. Cheeney, Wickersham, H. F. Smith, Wagner, Petersen, Schloetzer, Deniston, C. G. Smith, Davis, Amerman, Holmes and Bevan.

Under the head of proposals of new members, Dr. S. Faircloth was proposed by Drs. Wickersham and Bevan. Elected.

Dr. Wickersham then read a paper on a case of small pox and measles occurring simultaneously in the same patient. The case corroborates the statement of various observers, that the two diseases occurring together in the same patient, the measles first runs its course, the variolous disease being rendered latent or held in abeyance (although the prodromic symptoms may have been those of small pox) to proceed in development and desquamation after the rubeola has disappeared.

After the discussion of Dr. W.'s paper, Dr. Petersen had the floor, and read a portion of a paper commenced at the last meeting, on the Etiology, Symptoms and Prognosis of Hemorrhoids.

In the order of reports, Dr. N. S. Davis, Chairman of Com. on Prize Essays, presented a report and resolutions, which were accepted and adopted.

On motion, Drs. Davis, Fisher and Cheeney were appointed a committee to decide upon the merits of the essays offered, and award the prize.

A committee was then appointed, of which Dr. Schloetzer is chairman, and Drs. Chas. G. Smith and Amerman members, to report a subject at the next meeting on which the essays should be written.

Dr. Davis made a verbal report on the sanitary condition of the city: had observed some cholera morbus in children, one case of spasmodic cholera in an adult, since the commencement of hot weather; also the prevalence of diarrhœa of a remittent character, discharges copious, with more than usual periodicity, yielding readily to anti-periodics, added to the usual treatment. From the early appearance of this tendency, he predicted the prevalence of much periodic disease in the latter part of summer and autumn. Also reported a few cases of disease in children: the main phenomena presented were great difficulty of breathing, pale skin, sunken look of the face and eyes, feeble pulse, and rapid superficial respiration. The medication was expectorant, alterative and anti-periodic.

Dr. Petersen reported some remittents among immigrants; also typhoid diarrhœa; a tonic, anti-periodic therapeutic was instituted.

Drs. Wickersham and Cheeney reported cases of diarrhœa and remittent disease. Dr. C. had especially observed the prevalence of diarrhœa in the prison during the hot days of last week.

On motion, a special meeting was appointed two weeks from to-night, July 21st, at Dr. Davis' office, for the discussion of cholera infantum, Dr. L. P. Cheeney opening the discussion.

Adjourned.

THOMAS BEVAN, *Secretary.*

